# Supplementary material for: Expression of a Fungal Lectin in Arabidopsis Enhances Plant Growth and Resistance Toward Microbial Pathogens and a Plant-Parasitic Nematode
Source: Front Plant Sci. 2021 Apr 9;12:657451. doi: 10.3389/fpls.2021.657451 (PMC8063123; doi:10.3389/fpls.2021.657451)
Supplement: Supplementary Figure 1 — CCL2 expression in E. coli and purification. [file Data_Sheet_1.docx]

**Supporting information**

| **Table S1:** Sequences of primers for qPCR. F: Forward primer. R: Reverse primer. | |
| --- | --- |
| **Primer** | **Sequence (5’-3’)** |
| *PDF 1.2* (AT5G44420) | **F:**GTTCTCTTTGCTGCTTTCGAC  **R:**GCAAACCCCTGACCATGT |
| *PR-1* (AT2G14610) | **F:**ACTACAACTACGCTGCGAACAC  **R:**GTTACACCTCACTTTGGCACATC |
| *OBP2* (AT1G07640) | **F:**TATGTAAGCTCGCCACGATACGCT  **R:**AACACGATCAACCGGCTCTAACGA |
| *GLI1* (AT1G80460) | **F:** GTGCATTGCAAAGGCTCTCGACAA  **R:** AAGGCCAGTGGATTTGCTCCAAAC |
| *GLY1* (AT2G40690) | **F:**ACCTGCATGCTTAGACCTCCACTT  **R:**ACATTTGGAGAAGAAACGCAGCGG |
| *RBOHD* (AT5G47910) | **F:**GATCAAGGTGGCTGTTTACCC  **R:**TCGGCAGTTCACCAACATGA |
| *RBOHF* (AT1G64060) | **F:**TGACACGCCAAGACGAAAGA  **R:**ATCACACCCCGTTGGTCAAG |
| *expG* (AT4G26410) | **F:**GAGCTGAAGTGGCTTCCATGAC  **R:**GGTCCGACATACCCATGATCC |
| *oprF* (Genbank: 878442) | **F:**GTGTTCATCACAAGCGGCAT  **R:**GGGAAGCACCTGGAGTCAAT |
| *Cutinase A* (Genbank: Z69264) | **F:**AGCCTTATGTCCCTTCCCTTG  **R:**GAAGAGAAATGGAAAATGGTGAG |


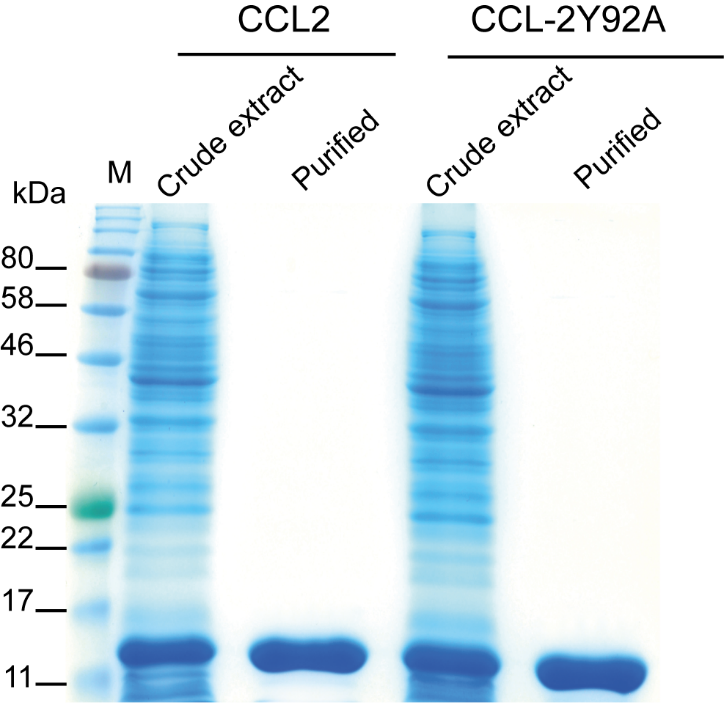


**Figure S1** CCL2 expression in *E. coli* and purification. SDS-PAGE of crude extract and purified protein fractions of CCL2 and CCL2-Y92A. M: protein size marker. The CCL2 proteins were extracted from *E. coli* 18h after IPTG-treatment. Purification was performed by Ni-NTA chromatography.

**
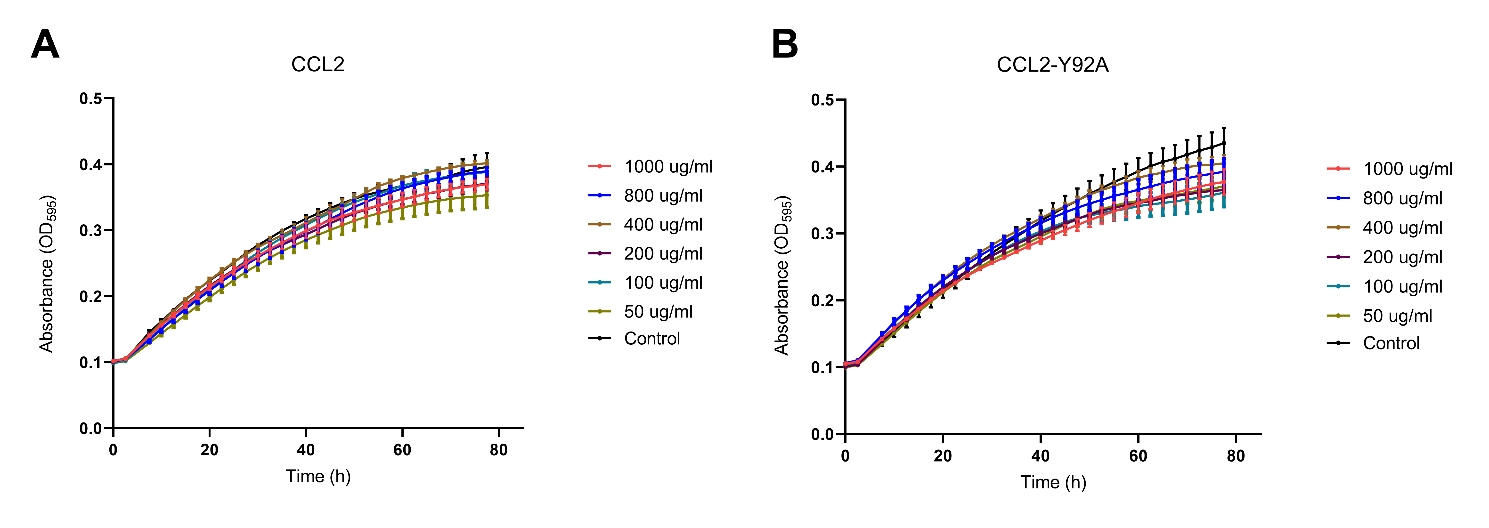
**

**Figure S2** *In vitro* antimicrobial assay of bacterially produced CCL2 and CCL2-Y92A against *B. cinerea*. 10^3^ spores mL^-1^ of *B. cinerea* were incubated with different concentrations (0-1000 µg/mL) of bacterially produced and purified CCL2 (A) and CCL2-Y92A (B) protein. Every four hours, the OD_595_ was measured using the cell imaging multi‐mode plate reader Cytation™ 5. Reads were plotted as a growth curve. Measurements are averages of four replicates. Error bars show the standard deviation.
